# Real-world cost-effectiveness of multi-gene panel sequencing to inform therapeutic decisions for advanced non-small cell lung cancer: a population-based study

**DOI:** 10.1016/j.lana.2024.100936

**Published:** 2024-11-15

**Authors:** Emanuel Krebs, Deirdre Weymann, Cheryl Ho, Ian Bosdet, Janessa Laskin, Howard J. Lim, Stephen Yip, Aly Karsan, Timothy P. Hanna, Samantha Pollard, Dean A. Regier

**Affiliations:** a Cancer Control Research, BC Cancer Research Institute, Vancouver, BC, Canada; b Department of Medical Oncology, BC Cancer, Vancouver, BC, Canada; c Department of Medicine, Faculty of Medicine, University of British Columbia, Vancouver, BC, Canada; d Department of Pathology & Laboratory Medicine, Faculty of Medicine, University of British Columbia, Vancouver, BC, Canada; e Cancer Genetics & Genomics Laboratory, BC Cancer, Vancouver, BC, Canada; f Michael Smith Genome Sciences Centre, BC Cancer Research Institute, Vancouver, BC, Canada; g Department of Oncology, Queen's University, Kingston, ON, Canada; h Department of Public Health Science, Queen's University, Kingston, ON, Canada; i School of Population and Public Health, Faculty of Medicine, University of British Columbia; Vancouver, BC, Canada

**Keywords:** Lung cancer, Genetic testing, Multi-gene panel sequencing, Cost-effectiveness, Real world data, Real world evidence, Targeted treatment

## Abstract

**Background:**

Multi-gene panel sequencing streamlines treatment selection for advanced non-small cell lung cancer (NSCLC). Implementation continues to be uneven across jurisdictions, partly due to uncertain clinical and economic impacts. In British Columbia (BC), Canada, the public healthcare system reimbursed a multi-gene panel in September 2016. This study determined the population-level cost-effectiveness of publicly reimbursed multi-gene panel sequencing compared to single-gene testing for advanced NSCLC.

**Methods:**

Our population-based retrospective study design used patient-level linked administrative health databases. We considered adult BC residents with a panel-eligible lung cancer diagnosis between September 2016 and December 2018. Using a machine learning approach, we conducted 1:1 genetic algorithm matching of recipients receiving multi-gene panel sequencing to controls receiving single-gene testing, maximising balance on observed demographic and clinical characteristics. Following matching, we estimated mean three-year survival time and costs (public healthcare payer perspective; 2021 CAD) and calculated the incremental net monetary benefit (INMB) for life-years gained (LYG) at conventional willingness-to-pay thresholds using inverse probability of censoring weighted linear regression and nonparametric bootstrapping.

**Findings:**

We matched 858 panel-eligible advanced NSCLC patients to controls, achieving balance for the 16 included covariates. Average test turnaround times were 18.6 days for multi-gene panel sequencing and 7.0 days for single-gene testing. After matching, mean incremental costs were $3529 (95% CI: −$4268, $10,942) and mean incremental LYG were 0.08 (95% CI: −0.04, 0.18). Among the 1000 bootstrap samples, 14.5% had lower costs and increased survival and 78.6% had higher costs and increased survival. The INMB was $523 (95% CI: −$6256, $7023) at $50,000/LYG, with a 57.5% probability of being cost-effective, and $4575 (95% CI: −$5468, $14,064) at $100,000/LYG, with an 84.0% probability of being cost-effective.

**Interpretation:**

Using population-based real-world data, we found a moderate to high probability that panel-based testing to inform targeted treatment for NSCLC would be cost-effective at higher thresholds.

**Funding:**

This research was supported by 10.13039/501100000233Genome British Columbia/10.13039/100008762Genome Canada (G05CHS) and the 10.13039/501100004376Terry Fox Research Institute.

**Research in context**

**Evidence before this study**

Discovery of driver mutations and targeted therapies has resulted in national consensus recommendations to implement multi-gene panel sequencing for patients with advanced non-small cell lung cancer (NSCLC). National recommendations for the use of multi-gene panel sequencing do not include explicit considerations of cost-effectiveness, and implementation continues to be uneven across jurisdictions, in part due to uncertain clinical and economic impacts. For reports on the cost-effectiveness of multi-gene panel sequencing for patients with advanced NSCLC, we searched PubMed on May 17, 2024, for papers published since inception using the search terms “lung cancer AND (multigene panel OR genetic testing) AND cost effectiveness”, without any language restrictions. We narrowed the search results to include reports of cost-effectiveness in NSCLC. All studies but one used simplistic decision analytic models, mostly decision trees, and all but two were predominantly informed by data generated from published literature or short-term targeted treatment trials that do not align with real-world patient outcomes. The single report drawing from a large nationwide database not based on a simplistic decision tree model used simulated patients to determine the cost-effectiveness of parallel next generation sequencing compared to single-gene–based sequential testing. Findings of increased quality-adjusted life years (0.12) and increased costs (€8357) from this model-based report were considered to be cost-effective according to national thresholds. We also searched PubMed for additional reports using real-world data for patients with advanced NSCLC using the search terms “lung cancer AND (multigene panel OR genetic testing) AND (real world data OR real-world evidence)” and found no additional articles on the cost-effectiveness of multi-gene panels. We did not find any cost-effectiveness reports offering evidence regarding the decision uncertainty related to the real-world implementation of multi-gene panels.

**Added value of this study**

This report on the real-world cost-effectiveness of multi-gene panel sequencing implementation is, to our best knowledge, the first to use population-level real world data for patients with advanced non-small cell lung cancer. With the use of machine learning-based quasi-experimental methods, we found a moderate to high probability that panel-based testing would be cost-effective at higher thresholds even with differences in survival and costs that were not statistically significant compared to single-gene testing. We quantify the uncertainty associated with diverse patient populations and contribute a nuanced perspective of multi-gene panel implementation.

**Implications of all the available evidence**

Our results show that multi-gene panel sequencing alone does not result in statistically significant survival differences or changes in cost, yet we found a high proportion of instances resulting in improved survival with a high probability multi-gene panel testing would be cost-effective at greater willingness-to-pay thresholds. Absence of an independent association of multi-gene panel sequencing with improved survival remained consistent with previous reports. The use of real-world evidence is important to provide clinically realistic findings. Our findings can be used to inform priority setting regarding policy of implementing multi-gene panel sequencing for advanced non-small cell lung cancer.

## Introduction

The development of treatments that target genetic mutations driving cancer growth has changed the care trajectory for patients with advanced non-small cell lung cancer (NSCLC), improving expected survival and quality of life.^1^, ^2^, ^3^ With the use of appropriate molecular tests, targetable mutations can be observed in up to 50% of patients with NSCLC.^4^ Mutated forms of the epidermal growth factor receptor (EGFR) gene were the first targets for tyrosine kinase inhibitors (TKIs) in NSCLC.^5^^,^^6^ Targeting EGFR mutations with TKIs has become standard care, offering greater tolerance in addition to improved outcomes compared to chemotherapy.^7^ Single-gene companion diagnostic tests can be used to detect EGFR mutations but discovery of driver mutations and targeted therapies has resulted in national consensus recommendations to implement multi-gene panel sequencing for optimizing clinical care.^8^^,^^9^

Panel-based sequencing targeting multiple genes simultaneously can streamline treatment selection for NSCLC by identifying patients for standard-of-care targeted therapies or facilitating access to novel treatments, clinical trials and compassionate access programs. Multi-gene panels have the potential of being cost- and time-effective by reducing the need for sequential single-gene testing, which may be linked to additional biopsies resulting in increased costs, patient burden, and lengthening diagnostic timelines. Additionally, panels can reduce the need for investments in multiple analytical platforms. In September 2016, British Columbia (BC) was the first Canadian healthcare system to reimburse multi-gene panel sequencing for patients with advanced NSCLC. BC implemented Oncopanel, an in-house lab developed next-generation sequencing panel capable of identifying genetic variants in 54 genes simultaneously to provide predictive, prognostic, and diagnostic information.^10^^,^^11^ The real-world economic impacts of Oncopanel implementation have not been assessed^12^ and BC remains Canada's only province with publicly reimbursed standard of care multi-gene panel sequencing for all newly diagnosed patients with advanced NSCLC.^13^

A recent systematic review on the cost-effectiveness of molecular tests for targeted advanced NSCLC therapies found only one publication directly comparing single-gene testing to multi-gene panel sequencing.^14^^,^^15^ Results from recent industry-supported model-based economic evaluations have projected meaningful survival benefits and considerable value for money for multi-gene panels compared to single-gene testing.^16^, ^17^, ^18^, ^19^, ^20^, ^21^, ^22^, ^23^ Most studies used simplistic decision analytic models, and all but two were based on published data generated from randomised control trials for targeted treatments composed of highly selected patient populations.^14^, ^15^, ^16^, ^17^, ^18^, ^19^, ^20^, ^21^, ^22^, ^23^

National recommendations for the use of multi-gene panel sequencing to optimise clinical management of advanced NSCLC do not include explicit considerations of cost-effectiveness,^24^^,^^25^ and multi-gene panel sequencing continues to be underutilised.^26^ The limited evidence that exist on the real-world cost-effectiveness of population-level implementation of multi-gene panel sequencing for patients with advanced NSCLC emphasises the need for real-world evidence (RWE) on the effectiveness and cost-effectiveness of multi-gene panels.^14^^,^^15^ We determined the cost-effectiveness of publicly reimbursed multi-gene panel sequencing compared to single-gene EGFR testing for all patients with advanced NSCLC in British Columbia, Canada.

## Methods

### Study setting and data sources

This retrospective study considered adult BC residents who had a non-neuroendocrine advanced (stage IIIB/IV) NSCLC diagnosis between September 1, 2016 and December 31, 2018. BC has single-payer, universal healthcare access for all residents and this study was based on a patient-level linkage of health administrative databases provided by BC Cancer and Population Data BC (Supplement S1). This study was approved by the University of British Columbia-BC Cancer Research Ethics Board (H20-00861).

### Study cohort

We used diagnosis records from the BC Cancer Registry and testing records from the BC Cancer Genetics and Genomics Lab to define the study cohort. Our study included all adult BC patients with an advanced NSCLC diagnosis and who received multi-gene panel sequencing or single-gene EGFR testing during the study period. Patients receiving multi-gene panel sequencing at any time during the study period were included in the multi-gene panel group, irrespective of whether they received single-gene testing in addition to multi-gene panel sequencing. The index date for study inclusion was based on the date the sequencing lab received the biopsy sample. Patients were excluded from the study cohort if linkage to health administrative databases was missing or if they died before the lab received the biopsy sample. We observed all patients included in our study cohort for a maximum follow-up period of three years or up to the first of either date of death, last date of enrolment in MSP, or December 31, 2019 (for a minimum observation period of one year for patients alive at end of follow-up).

### Derived variables and matching

We derived key covariates hypothesised to influence costs and to correlate with treatment outcomes, including patient demographics, clinical characteristics, and treatment histories at the index date (Supplement S1).

We matched patients receiving multi-gene panel sequencing 1:1 with contemporaneous control patients receiving single-gene testing to address confounding. We used genetic algorithm-based matching, a machine-learning approach that automates the process of maximising balance on observed covariates.^27^ This method is a form of nearest-neighbour matching where distances used to match patients are based on a generalised weighted Mahalanobis distance metric rather than a difference in parametrically estimated propensity scores. The genetic search algorithm converges on the optimal set of weights for each covariate to minimize differences in covariates after matching according to a pre-specified optimization criterion. We defined this criterion as maximising p-values from bootstrapped Kolmogorov–Smirnov tests in continuous variables and paired *t-tests* in binary variables, using lexical optimisation for a fixed sample size.^28^ We included a propensity score during genetic matching, as recommended to provide starting values for the genetic evolutionary search algorithm.^27^ We estimated this propensity score using logistic regression.

We prioritised covariates for matching based on clinician input and greatest imbalance at baseline, using a directed acyclic graph (DAG) for identifying covariates that must be measured and controlled to obtain an unconfounded effect estimate (Supplement S1).^29^^,^^30^ We performed exact matching on the three non-continuous covariates with the greatest imbalance (i.e., number of prior lines of systemic therapy, history of surgical treatment, and residency in the Vancouver Coastal Health Authority). Exact matching is a form of stratum matching where minimising the differences in covariates takes place within each exact matching stratum. We selected matches to maximise the balance of baseline covariates across patients receiving multi-gene panel sequencing and single-gene testing, allowing for ties and matching without replacement. We compared balance across matched and unmatched patients using standardised mean differences and variance ratios, including balance assessment on polynomial and interaction terms, and determined satisfactory balance as achieving a matched sample where all standardised mean differences were less than 0.1 and where variance ratios were less than 2.^28^ We performed genetic matching using the Matching package in R.^27^^,^^28^

### Effectiveness and cost-effectiveness

Overall survival time was determined from index date to death or censoring. Patients were censored if they were alive at the end of the observation period, or if they were no longer registered for universal healthcare access in BC through MSP. Following matching, we estimated the effect of multi-gene panel sequencing on overall survival using Weibull regression and Kaplan–Meier survival analysis. For the cost-effectiveness analysis, we calculated mean cost and survival over a maximum period of three years, divided into monthly intervals. We adhered to best-practice guidelines for the reporting of health economic evaluation and reported both costs and life-years gained (LYG) using a 1.5% annual discount rate.

#### Costs

We measured all healthcare expenditures from a public healthcare payer perspective using health resources use identified from the linked health administrative databases, including hospitalisation costs, day surgeries, physician services, publicly reimbursed outpatient prescription drugs, and cancer care. Cancer care included costs for systemic therapy, radiation therapy, medical appointments, and testing (Supplement S2). Costs per test to the lab were $1200 for the multi-gene panel and $228 for single-gene EGFR testing, including costs of reagents, extraction, sequencing, and labour. Costs for all other lab tests received by the study cohort were also included. Costs were adjusted to 2021 Canadian dollars using the medical care component of the Consumer Price Index.

#### Net Monetary Benefit (NMB) regression analysis

After matching, we weighted costs and survival time based on the inverse probability of being observed at the start of each monthly interval to account for censoring arising from incomplete follow-up data. We then applied inverse probability of censoring weighted linear regression to estimate mean three-year survival time and costs, in which the weights are summed in each interval to produce a weighted total. Finally, we used cost and effectiveness outcomes to calculate the incremental net monetary benefit (INMB) for life-years gained (LYG) at commonly reported willingness-to-pay thresholds of $50,000/LYG and $100,000/LYG. We used non-parametric bootstrapping to simulate sampling distributions for cost and survival and allowed confidence intervals to account for the correlation between costs and effectiveness observed in the data. We calculated bias-corrected 95% confidence intervals with 1000 replications.

### Sensitivity analysis

We conducted sensitivity analysis on several components of our analysis to assess the robustness of findings. First, we explored results sensitivity to our matching specification by matching on the same variables as the primary analysis except for (i) matching with replacement, restricting each control to be used a maximum of three times, (ii) no exact matching performed on region, and exact matching on additional regions (iii) Interior Health Authority and (iv) Fraser Health Authority. Second, we considered sensitivity to the price of multi-gene panel testing and the perspective of our costing analysis, by using the cost of single-gene testing for multi-gene panel sequencing, and conducting our analysis from a societal perspective, including out-of-pocket payments for prescription drugs. Third, we examined sensitivity to clinical information obtained from testing by conducting subgroup analyses based on positive or negative test results for EGFR mutation status. Finally, we assessed sensitivity to a positive correlation between end-of-life healthcare costs and censoring by estimating the cost-effectiveness over one- and two-year study periods and employing the seemingly unrelated regression (SUR) estimation approach to estimate mean three-year survival time and costs.

All analyses were done using Stata (version 16.1) and R (version 4.3.1).

### Role of the funding source

The funder of the study had no role in study design, data collection, data analysis, data interpretation, or writing of the report.

## Results

**Fig. 1:**
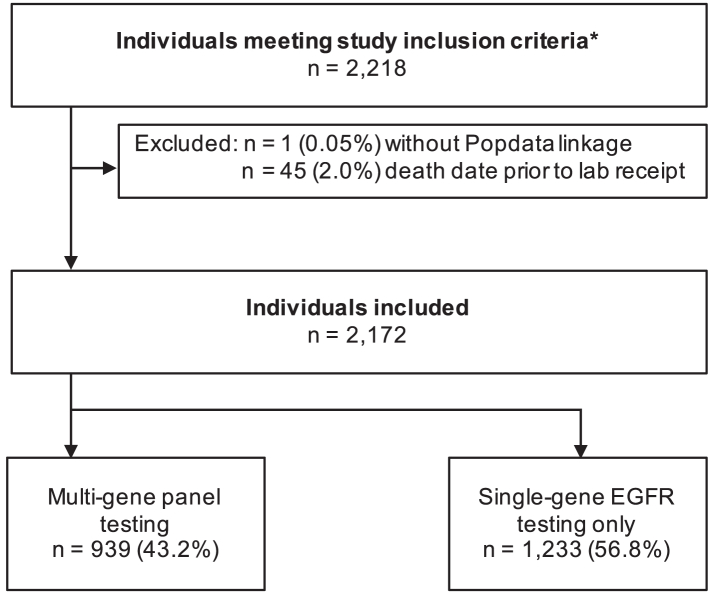
**Study flow diagram.** ∗ BC residents aged 18 years and older who had an advanced non-small cell lung cancer diagnosis between September 1, 2016 and December 31, 2018 and received multi-gene panel sequencing or single-gene epidermal growth factor receptor (EGFR) testing.

During our study period, there were 2218 adult BC residents who had a panel-eligible advanced NSCLC diagnosis and received either multi-gene panel sequencing or single-gene testing (Fig. 1). We excluded 45 patients (2.0%) from our analysis with a date of death before the lab received their biopsy sample, and one patient without linkage to health administrative databases. Among the 2172 patients included in our analysis, the average age was 68.9 years old (standard deviation: 10.2), a majority were women (54.8%; n = 1190), most had a single cancer site identified at the time of diagnosis (68.8%; n = 1495), and most had a cancer-related hospitalisation during the previous twelve months (53.8%; n = 1169). Over the study period, 939 patients (43.2%) received multi-gene panel testing and 1233 patients (56.8%) received single-gene EGFR testing. Of the patients included in the multi-gene panel testing group, 23 (2.5%) received a single-gene test before the multi-gene panel and six (0.6%) received a single-gene test following the multi-gene panel.

Table 1 summarises the demographic and clinical characteristics of the unmatched and matched patients. Compared to unmatched control patients, patients receiving multi-gene panel sequencing were younger (68.0 versus 69.6 years old) and received testing later from the time of diagnosis (mean 188 versus 136 days). Furthermore, a higher proportion of multi-gene panel patients had received one or more lines of systemic therapy (19.7% versus 13.6%) or surgical treatment (12.9% versus 5.6%) prior to testing, and resided within the Vancouver Coastal Health Authority (26.2% versus 16.3%) or in a Metropolitan area (52.1% versus 46.4%). Average turnaround times for receiving test results were 18.6 days for multi-gene panel sequencing and 7.0 days for single-gene testing.

**Table 1:** Baseline patient characteristics by test type received, before and after matching.

	Unmatched			Matched		
	Single-gene	Multi-gene panel	SMD	Single-gene	Multi-gene panel	SMD
	n (%)	n (%)		n (%)	n (%)	
No.	1233 (100.0)	939 (100.0)		858 (100.0)	858 (100.0)	
Sex						
Female	677 (54.9)	513 (54.6)	0.006	467 (54.4)	469 (54.7)	0.005
Male	556 (45.1)	426 (45.4)		391 (45.6)	389 (45.3)	
Age at diagnosis (years), mean (SD)	69.6 (10.2)	68.0 (10.2)	0.154	68.5 (10.1)	68.2 (10.1)	0.031
Times since diagnosis (days), mean (SD)^a^	136 (283)	188 (332)	0.169	143 (289)	154 (293)	0.039
Times since diagnosis >1 year^a^	126 (10.2)	162 (17.3)	0.205	99 (11.5)	115 (13.4)	0.056
Number of cancer sites at diagnosis						
1	853 (69.2)	642 (68.4)	0.047	590 (68.8)	594 (69.2)	0.048
2	271 (22.0)	201 (21.4)		194 (22.6)	181 (21.1)	
3 or more	109 (8.8)	96 (10.2)		74 (8.6)	83 (9.7)	
Number of prior lines of systemic therapy^b^						
0	1065 (86.4)	754 (80.3)	0.164	715 (83.3)	715 (83.3)	<0.001
1	147 (11.9)	162 (17.3)		129 (15.0)	129 (15.0)	
2 or more	21 (1.7)	23 (2.4)		14 (1.6)	14 (1.6)	
Any prior surgery^b^	69 (5.6)	121 (12.9)	0.254	69 (8.0)	69 (8.0)	<0.001
Any prior radiotherapy with palliative intent	356 (28.9)	241 (25.7)	0.072	239 (27.9)	220 (25.6)	0.050
Any cancer-related hospitalization, L12M	657 (53.3)	512 (54.5)	0.025	471 (54.9)	481 (56.1)	0.023
Modified CCI score >0	263 (21.3)	181 (19.3)	0.051	169 (19.7)	165 (19.2)	0.012
CDS > median value among females	376 (30.5)	231 (24.6)	0.132	224 (26.1)	207 (24.1)	0.046
CDS > median value among males	304 (24.7)	214 (22.8)	0.044	194 (22.6)	192 (22.4)	0.006
Geographic region						
Metropolitan	572 (46.4)	489 (52.1)	0.114	429 (50.0)	433 (50.5)	0.009
Urban	463 (37.5)	332 (35.4)	0.046	307 (35.8)	317 (36.9)	0.024
Rural	198 (16.1)	118 (12.6)	0.100	122 (14.2)	108 (12.6)	0.048
Provincial health authority						
Interior	221 (17.9)	187 (19.9)	0.051	183 (21.3)	179 (20.9)	0.011
Fraser	433 (35.1)	285 (30.4)	0.102	275 (32.1)	271 (31.6)	0.010
Vancouver Coastal^b^	201 (16.3)	246 (26.2)	0.244	198 (23.1)	198 (23.1)	<0.001
Island	307 (24.9)	182 (19.4)	0.133	162 (18.9)	172 (20.0)	0.029
Northern	71 (5.8)	37 (3.9)	0.085	40 (4.7)	36 (4.2)	0.023
Health resource use costs, LM, median [IQR]	2780 [844; 8164]	2811 [718; 7324]	0.046	2706 [801; 7378]	2880 [789; 7594]	0.012

CCI: Charlson Comorbidity Index (CCI) calculated for the L12M using ICD-10 codes from hospitalization records that excluded exclude cancer-specific ICD-10 codes.

CDS: Chronic Disease Score based on dispensation records of 29 non-cancer prescription medication categories for L6M; SMD: Absolute standardized mean difference.

Provincial Health Authority: health delivery areas within the province of BC; SD: Standard deviation; LM: Last month; L6M: Last six months; L12M: Last twelve months.

a Time elapsed between date of initial diagnosis and date when the lab received their biopsy sample for testing.

b Exact matching covariates.

After matching, a total of 858 (91.4%) multi-gene panel sequencing patients were matched to single-gene tested patients. Matching achieved good balance with standardized differences <0.1 for all covariates (Supplement S3), with a maximum variance ratio of 1.25 for the number of cancer sites at diagnosis, and a maximum bootstrapped Kolmogorov–Smirnov test statistic of 0.075 (p-value = 0.022) for time since diagnosis (see Supplement S4 for Quantile–Quantile plot).

In the final matched cohort, the proportion of positive EGFR mutation findings were equivalent (20.9% for multi-gene panels versus 20.5% for single-gene), whereas proportions of negative findings (74.9% for multi-gene panels versus 64.6%) and inconclusive EGFR results (4.2% for multi-gene panels versus 14.9% for single-gene) differed (p-value < 0.001) (Supplement S5). Among patients with positive findings, the proportion receiving TKIs targeting EGFR mutations were comparable (72.6% for multi-gene panels versus 80.7% for single-gene; p-value = 0.10). Receipt of any systemic therapy among patients with negative EGFR findings was lower but comparable across groups (49.8% for multi-gene panels versus 46.6% for single-gene; p-value = 0.30).

**Fig. 2:**
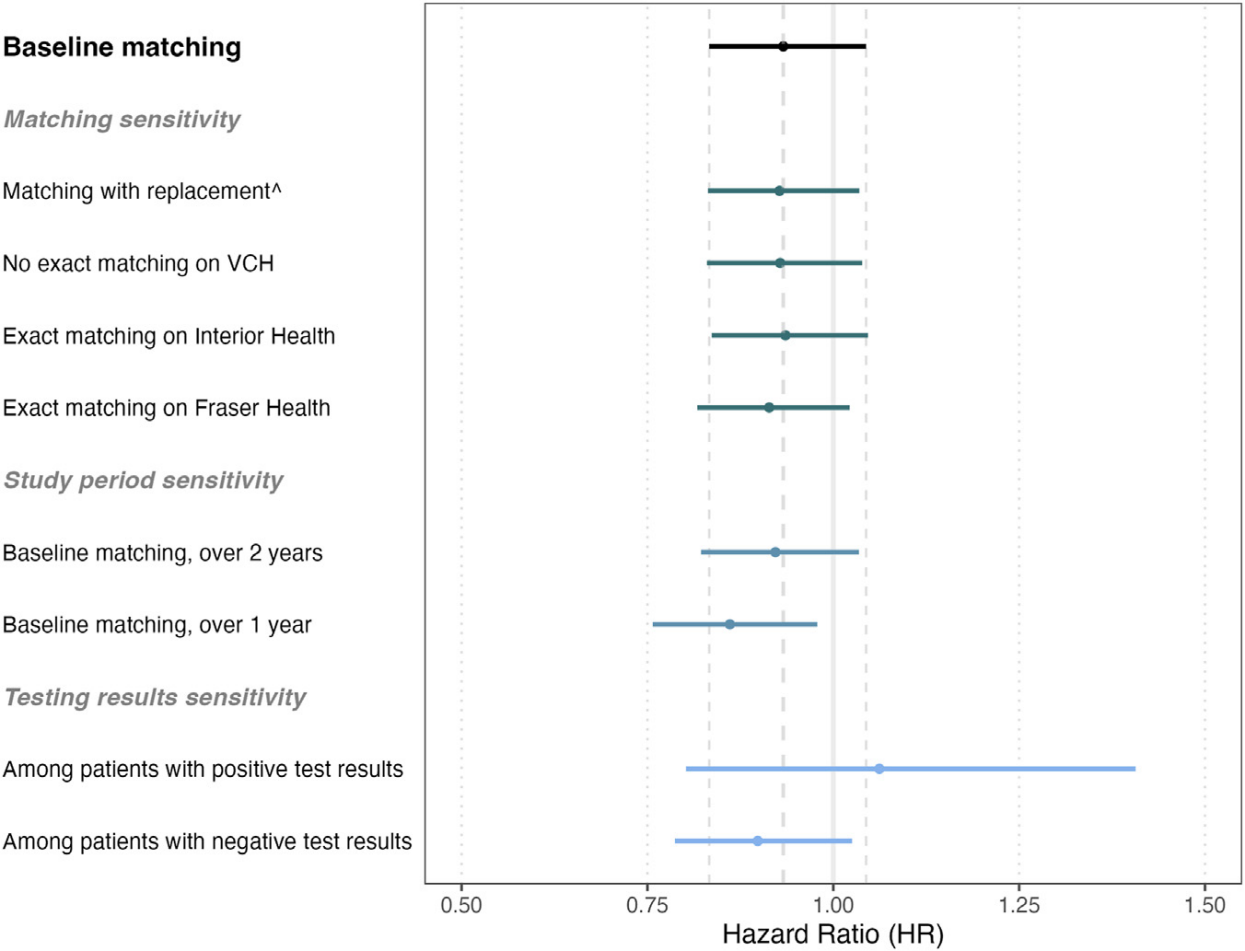
**Estimated effect on overall survival of receiving multi-gene panel sequencing compared to single-gene testing.** The effect of multi-gene panel sequencing on overall survival was estimated using Weibull regression, with HR <1 indicating an effect on overall survival favoring multi-gene panel sequencing. VCH (Vancouver Coastal Health), Fraser Health, and Interior Health are health delivery areas within the province of BC. ˆMatching with replacement was restricted to using each control a maximum of three times.

Differences on overall survival of multi-gene panel sequencing compared to single-gene testing were not statistically significant using either Weibull regression (Hazard Ratio: 0.932 [95% CI: 0.833–1.044]; Fig. 2) or the Kaplan–Meier estimate of the survivor function (Log rank test p-value: 0.16; Supplement S6). From the Kaplan–Meier survival analysis, mean survival time for matched patients receiving multi-gene panel sequencing was 469 days (95% CI: 440–497) and 442 days (95% CI: 414–471) for matched single-gene tested patients (Supplement S7). Correspondingly, median survival time for matched patients receiving multi-gene panel sequencing was 325 days (95% CI: 279–384) and 258 days (95% CI: 223–306) for matched single-gene tested patients (Supplement S8). The proportion of censored observations were 31.7% and 26.1% among matched multi-gene panel sequencing and single-gene patients, respectively.

**Fig. 3:**
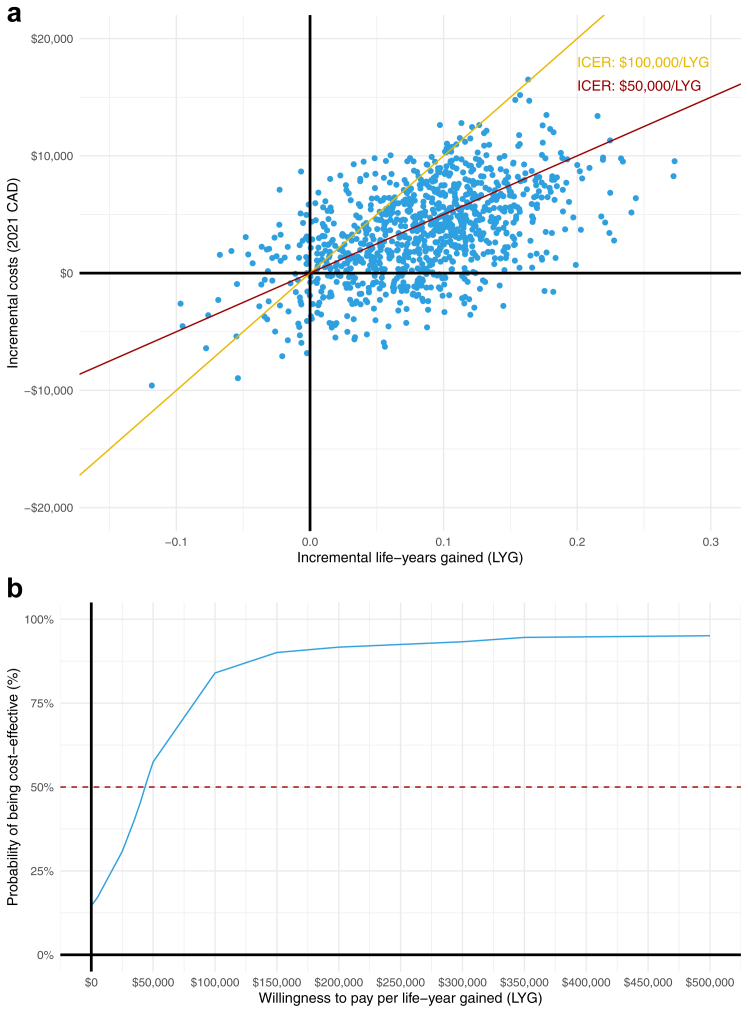
**Cost-effectiveness plane (panel a) and cost-effectiveness acceptability curve (panel b) for multi-gene panel testing compared to single-gene EGFR testing.** The points in panel a represent the 1000 non-parametric bootstrap simulations and indicate incremental LYG and incremental costs of multi-gene panel sequencing versus single-gene testing. The cost-effectiveness plane is divided into four quadrants with the north-east (NE) quadrant representing increased survival and higher costs. Points below the lines represent cost-effective simulations at the indicated thresholds. The cost-effectiveness acceptability curve (CEAC) in panel b summarizes the uncertainty from the non-parametric bootstrap simulations in relation to a range of willingness to pay thresholds with the vertical axis representing the probability that multi-gene panel sequencing would be cost-effective.

Incremental costs of multi-gene panel sequencing compared to single-gene testing were $3529 (95% CI: −$4268, $10,942) (Table 2). Similarly, the estimate for incremental life-years gained was 0.08 (95% CI: −0.04, 0.18). Among the 1000 bootstrap samples, 14.5% had lower costs and increased survival and 78.6% had higher costs and increased survival (Fig. 3). The probability of multi-gene panel sequencing being cost-effective was 57.5% at $50,000/LYG and 84.0% at $100,000/LYG (Fig. 3). Systemic therapy was the largest cost category for both multi-gene panels testing (49.2%) and single-gene testing (52.4%) (Supplement S8). The largest difference in costs between multi-gene panel sequencing and single-gene testing was for inpatient and outpatient care $1593 (95% CI: −$829, $4014), mostly accrued during the first year of the study period (Supplement S8).

**Table 2:** Cost-effectiveness of multi-gene panel sequencing compared to single-gene testing for advanced non-small cell lung cancer (NSCLC)^a^.

	Treated (*n*)^b^	Incremental life-years gained (LYG)	Incremental total cost, 2021 CAD	INMB, $50,000/LYG	% C-E	INMB, $100,000/LYG	% C-E
**Base case**	858	0.08 [−0.04, 0.18]	$3529 [−$4268, $10,942]	$523 [−$6256, $7023]	57.5%	$4575 [−$5468, $14,064]	84.0%
Sensitivity analysis							
Matching with replacement^c^	932	0.09 [−0.01, 0.21]	$3942 [−$3143, $11,639]	$455 [−$6024, $6942]	54.4%	$4851 [−$4817, $14,465]	83.9%
Matching without VCH	884	0.08 [−0.02, 0.18]	$1456 [−$5965, $8829]	$2606 [−$4761, $8896]	73.7%	$6668 [−$2487, $15,466]	90.3%
Matching with Interior Health	884	0.07 [−0.05, 0.18]	$1360 [−$6675, $9557]	$2362 [−$4801, $9409]	69.4%	$6083 [−$4161, $15,756]	86.8%
Matching with Fraser Health	882	0.10 [−0.02, 0.21]	$4224 [−$3587, $11,620]	$592 [−$6203, $7880]	56.4%	$5409 [−$5406, $14,758]	86.4%
Reduced testing costs	858	0.08 [−0.04, 0.18]	$2548 [−$5244, $9962]	$1503 [−$5275, $8002]	66.4%	$5555 [−$4487, $15,044]	88.5%
Societal perspective	858	0.08 [−0.04, 0.18]	$4248 [−$3526, $12,202]	−$196 [−$7285, $6614]	47.6%	$3856 [−$5911, $13,239]	80.5%
Base, 2 years FU	858	0.07 [−0.01, 0.14]	$3004 [−$1617, $7793]	$587 [−$4,120, $4695]	63.8%	$4178 [−$2044, $10,608]	91.7%
Base, 1 year FU	858	0.04 [0.00, 0.08]	$2349 [−$146, $5183]	−$304 [−$3,164, $2235]	45.7%	$1741 [−$2236, $5578]	85.9%
Positive test results	179^d^	−0.01 [−0.27, 0.24]	$6612 [−$10,778, $25,392]	−$7294 [−$28,859, $13,313]	21.3%	−$7977 [−$28,859, $13,313]	35.7%
Negative test results	643^d^	0.11 [−0.04, 0.25]	$5223 [−$3624, $14,363]	$246 [−$8609, $8848]	51.3%	$5715 [−$6444, $18,752]	82.3%

INMB: Incremental net monetary benefit, calculated as: (incremental life-years gained X willingness to pay threshold)–incremental costs; INMB >0 indicating cost-effectiveness.

% C-E: percentage of 1000 botstrap samples found to be cost-effective; VCH: Vancouver Coastal Health.

a 95% confidence intervals are bias-corrected over 1000 bootstrapped samples; costs are presented from a publicly-funded health care sector perspective.

b Number of patients included in the analysis after 1:1 matching to controls.

c Matching with replacement was restricted to using each control a maximum of three times.

d Stratification on test results occurred after matching; there were 176 and 554 controls included in the positive and negative groups, respectively.

In sensitivity analyses considering alternative matching specifications, reduced multi-gene testing costs and shorter study periods, we found slightly higher probabilities of multi-gene panels being cost-effective at a $100,000/LYG threshold, although these results were characterized by small variations in incremental survival benefits and costs that remained not statistically significantly different from no difference (Table 2 & Supplement S9). Subgroup analysis according to test results indicated that while incremental costs were greatest among matched patients with positive test results ($6612; 95% CI: −$10,778, $25,392), although not statistically significantly different from no difference, incremental survival was lowest (−0.01; 95% CI: −0.27, 0.24). Reporting of our economic evaluation in accordance with best-practice guidelines are presented in Supplements S10–S12.

## Discussion

Our study estimated the real-world cost-effectiveness of publicly reimbursed multi-gene panel sequencing compared to single-marker EGFR testing for informing therapeutic decisions for advanced NSCLC. Previous model-based studies examining the cost-effectiveness of multi-gene versus single-gene testing have reported substantial improvement in survival outcomes when modelling the implementation of multi-gene panel sequencing,^16^, ^17^, ^18^, ^19^, ^20^, ^21^, ^22^ with several also reporting reduced costs to healthcare systems. Using RWD with a quasi-experimental study design, we found that multi-gene panel sequencing alone does not result in statistically significant survival differences (ΔLYG; 0.08; 95% CI: −0.04, 0.18) or changes in cost (ΔC: $3529; 95% CI: −$4268, $10,942) yet we found a high probability multi-gene panel testing would be cost-effective at greater willingness-to-pay thresholds compared to single-gene EGFR testing (84.0% at $100,000/LYG). Our findings additionally indicate that the cost-effectiveness of multi-gene panel sequencing was not primarily influenced by the sequencing cost alone.

Absence of an independent association of multi-gene panel sequencing with improved survival, in part due to low eligibility for TKIs targeting EGFR mutations, is consistent with previously reported RWE.^31^^,^^32^ Similarly, the potential association of multi-gene panel sequencing with improved survival for EGFR wild-type patients we found may imply that detection of other alterations can inform more effective treatment options, which is also consistent with prior findings.^32^ Our findings quantified the inherent uncertainty associated with diverse patient populations and we found a high proportion of instances resulting in improved survival, thereby contributing a nuanced perspective on the potential effectiveness and cost-effectiveness of multi-gene panel implementation. The landscape of treatment options for patients with advanced NSCLC has been rapidly changing since the end of our study period.^33^ Combined with the lower treatment receipt among patients with negative EGFR findings we observed, real-world evidence on the population-level impact of targeted therapies for patients harbouring non-EGFR mutations (e.g., ALK, ROS1 or NTRK) would be likely to capture additional benefits and value of multi-gene panel sequencing.^34^

Our RWE contrasts with recently published model-based economic evaluations that rely on simplistic decision tree models^15^, ^16^, ^17^, ^18^, ^19^, ^20^ predominantly informed by data generated from short-term randomised control trials evaluating targeted treatments featuring narrow study cohort definitions that may not align with real-world patient populations.^16^, ^17^, ^18^, ^19^, ^20^, ^21^, ^22^, ^23^ Exclusion of downstream health resource use costs that can result from next-generation sequencing^16^^,^^18^^,^^20^^,^^23^ with assumptions of increased detection of patients with an identified mutation and optimistic uptake projections for targeted therapies^16^, ^17^, ^18^, ^19^^,^^21^^,^^23^ may produce clinically unrealistic findings. Such approaches fail in providing evidence to reliably inform value for money for multi-gene panel sequencing for advanced NSCLC.

Another important aspect of the research to date^14^^,^^16^, ^17^, ^18^, ^19^, ^20^, ^21^, ^22^, ^23^ is that a majority appears to be directly supported by the pharmaceutical industry. Industry-supported research has been associated with more favourable efficacy results^35^ and more favourable ICER values across a range of disease areas^36^ and in oncology.^37^ Taken together, these findings suggest the generalisability of most published research on the cost-effectiveness of multi-gene panel sequencing compared to single-gene testing may be problematic, emphasising the importance of generating unbiased RWE from broad patient populations to inform decision-making.

The paucity of unbiased real-world evidence on the clinical benefit and cost-effectiveness of multi-gene panel sequencing alone is representative of a broader evidence generation challenge for precision oncology, which often lacks comparative evidence typically generated from randomised controlled trials.^38^ Our quasi-experimental study design supporting comparative causality and unbiased effect estimation provides the necessary information to fill the evidence gaps. The United States Food & Drug Administration, the European Medicines Agency as well as other national regulatory and reimbursement organizations have recognized the role RWE can play in reducing uncertainty, and have been formalizing RWE guidance. For RWE to reach its full potential and inform the adoption of precision oncology technologies offering good comparative value, the evidence must not only be of sufficient quality to be decision-grade but also needs to be accepted as such by decision-makers.^39^, ^40^, ^41^ In a continuously evolving landscape of precision oncology innovations, understanding how decision-makers evaluate compliance to RWE frameworks is of crucial importance for evidence generation to support implementation decisions and the sustainability of health systems.

Implementation of next-generation sequencing has been slow compared to the rapid development of new targeted therapies,^33^ partly driven by concerns regarding evidentiary uncertainty of patient-valued outcomes. The limited clinical value of multi-gene panel testing versus single-gene testing for advanced NSCLC found in this study corroborates these concerns. Projections suggest the potential for increased impacts for next-generation sequencing within the near future if more patients with actionable targets can be identified,^42^ yet increased uptake will require continued efforts to integrate broader patient needs and experiences.^43^^,^^44^ Patients understand that uncertainty around health benefits and clinical agreement on changing treatment based on test results are among the most important aspects of providing high-value genomics-based individualized care, and broad implementation of panel-based testing in healthcare systems will require for the evidence base to mature and uncertainty around implementation to be reduced.^45^^,^^46^ Continual assessment using RWD that integrates patient-valued outcomes can provide the opportunity to further our understanding of comparative effectiveness and value. A life-cycle health technology assessment framework embedded into learning healthcare systems can drive RWE generation to ultimately deliver on the tremendous potential of precision oncology innovation.^47^

### Limitations

This study had several limitations. First, while all measured covariates were well balanced across our matched cohort, there may be selection bias and residual confounding due to the observational nature of our study design. Critically, important factors in determining treatment selection and outcomes in cancer such as performance status are not routinely captured in health administrative databases. However, our use of machine learning-based quasi-experimental matching methods has been shown to outperform common propensity score matching,^48^ and we achieved balance on entire distributions of key covariates correlated with treatment outcomes, including several variables found in health administrative data that have been used to proxy performance status in cancer.^49^ Second, our study did not consider progression-free survival or other patient-reported outcomes such as health-related quality of life and the personal value of testing information itself, independent of clinical care.^43^ Given differences in survival that were not significant between multi-gene panel sequencing and single-gene testing, future studies will be necessary to determine whether the potential incremental benefits captured by other outcomes would increase the probability that broad implementation of multi-gene panel sequencing can provide additional value in healthcare systems. Third, time from biopsy sample collection may have resulted in a small number of biopsy samples with insufficient tumour content for multi-gene panel sequencing for which the lab would have overridden a multi-gene panel test request to perform single-gene testing. Nonetheless, our matching approach achieved balance on times from biopsy sample collection to testing across groups (Supplement S5), mitigating concerns of this factor confounding test selection or findings. Finally, while our study was based on comprehensive population-level RWD, given the nature of healthcare policies in BC, caution must be exercised when generalising our findings to other healthcare systems with different patient populations with different mutational prevalence and/or targeted treatment coverage.

### Conclusion

Our study demonstrates the importance of RWE to determine the economic value of multi-gene panel sequencing in advanced NSCLC. With the use of machine learning-based quasi-experimental methods, we identified a well-balanced counterfactual and found a high probability that panel-based testing would be cost-effective at higher thresholds even with differences in survival and costs that were not statistically significant compared to single-gene testing. These findings provide a nuanced perspective on the potential cost-effectiveness of multi-gene panel sequencing implementation and can be used to support healthcare systems’ deliberations on the broad implementation of panel-based genomic testing.

## Contributors

EK, DW, and DAR designed the study. EK wrote the first draft of the article. EK and DW executed the analysis. DAR, DW, CH, IB, JL, HJL, SY, AK, TPH, SP aided in the interpretation of results and provided critical revisions to the article. DAR secured funding for the study. EK and DW verified the underlying data. All authors approved the final draft. The corresponding author had full access to all the data in the study and had final responsibility for the decision to submit for publication.

## Data sharing statement

Access to data provided by the Data Steward(s) is subject to approval, but can be requested for research projects through the Data Steward(s) or their designated service providers. All inferences, opinions, and conclusions drawn in this publication are those of the authors, and do not reflect the opinions or policies of the Data Steward(s).

## Declaration of interests

The authors declare no competing non-financial interests but the following competing financial interests: D.A.R. has received travel funding from Illumina; his institution has received research funding for a project from Roche Canada. C.H. has received honoraria from Abbvie, Amgen, AstraZeneca, Bayer, BMS, Janssen, Jazz, Merck, Novartis, Pfizer, Roche and Sanofi; her institution has received research funding from AstraZeneca Canada and Roche Canada. D.W. & S.P. co-direct IMPRINT Research Consulting and have consulted for Roche Canada and AstraZeneca Canada. D.W. has also received travel funding from Illumina. S.Y. is a member of the advisory boards of and has received honoria from Amgen, AstraZeneca, Bayer, Incyte, Pfizer, Roche Canada and Servier. H.J.L. has received Honoria from Roche, Amgen, Pfizer, Eisai, Taiho, Astellas, Ipsen, BMS, and Merck for consultancy work. T.P.H. is the Ontario Health Cancer-Care Ontario (OH-CCO) Radiation Oncology Clinical Quality Lead for the Radiation Treatment Program.

## Appendix A Supplementary data

Oncopanel_Supplement

## References

[1] Geater S.L., Xu C.R., Zhou C. (2015). Symptom and quality of life improvement in LUX-Lung 6: an open-label phase III study of afatinib versus cisplatin/gemcitabine in Asian patients with EGFR mutation-positive advanced non–small-cell lung cancer. J Thorac Oncol.

[2] Chen G., Feng J., Zhou C. (2013). Quality of life (QoL) analyses from OPTIMAL (CTONG-0802), a phase III, randomised, open-label study of first-line erlotinib versus chemotherapy in patients with advanced EGFR mutation-positive non-small-cell lung cancer (NSCLC). Ann Oncol.

[3] Howlader N., Forjaz G., Mooradian M.J. (2020). The effect of advances in lung-cancer treatment on population mortality. N Engl J Med.

[4] Barlesi F., Mazieres J., Merlio J.P. (2016). Routine molecular profiling of patients with advanced non-small-cell lung cancer: results of a 1-year nationwide programme of the French Cooperative Thoracic Intergroup (IFCT). Lancet.

[5] Cheema P.K., Gomes M., Banerji S. (2020). Consensus recommendations for optimizing biomarker testing to identify and treat advanced EGFR-mutated non-small-cell lung cancer. Curr Oncol.

[6] Lindeman N.I., Cagle P.T., Beasley M.B. (2013). Molecular testing guideline for selection of lung cancer patients for EGFR and ALK tyrosine kinase inhibitors: guideline from the college of American pathologists, international association for the study of lung cancer, and association for molecular pathology. J Thorac Oncol.

[7] Mok T.S., Wu Y.L., Thongprasert S. (2009). Gefitinib or carboplatin–paclitaxel in pulmonary adenocarcinoma. N Engl J Med.

[8] Ettinger D.S., Wood D.E., Aisner D.L. (2023). NCCN guidelines® insights: non–small cell lung cancer, version 2.2023: featured updates to the NCCN guidelines. J Natl Compr Cancer Netw.

[9] Mosele F., Remon J., Mateo J. (2020). Recommendations for the use of next-generation sequencing (NGS) for patients with metastatic cancers: a report from the ESMO precision medicine working group. Ann Oncol.

[10] BC Cancer Foundation (2016). https://bccancerfoundation.com/news-and-media/blog/new-genetic-tests-become-standard-cancer-care-bc/.

[11] Cancer Genetics and Genomics Laboratory (2023). http://cancergeneticslab.ca/genes/oncopanel/.

[12] British Columbia Cancer Agency (2017). The Oncopanel Pilot (TOP) study. clinicaltrials.gov. NCT02171286.

[13] Yip S., Christofides A., Banerji S. (2019). A Canadian guideline on the use of next-generation sequencing in oncology. Curr Oncol.

[14] Henderson R., Keeling P., French D., Smart D., Sullivan R., Lawler M. (2021). Cost-effectiveness of precision diagnostic testing for precision medicine approaches against non-small-cell lung cancer: a systematic review. Mol Oncol.

[15] Steuten L., Goulart B., Meropol N.J., Pritchard D., Ramsey S.D. (2019). Cost effectiveness of multigene panel sequencing for patients with advanced non–small-cell lung cancer. JCO Clin Cancer Inform.

[16] Johnston K.M., Sheffield B.S., Yip S., Lakzadeh P., Qian C., Nam J. (2020). Comprehensive genomic profiling for non-small-cell lung cancer: health and budget impact. Curr Oncol.

[17] Lemmon C.A., Zhou J., Hobbs B., Pennell N.A. (2023). Modeling costs and life-years gained by population-wide next-generation sequencing or single-gene testing in nonsquamous non–small-cell lung cancer in the United States. JCO Precis Oncol.

[18] Sheffield B.S., Eaton K., Emond B. (2023). Cost savings of expedited care with upfront next-generation sequencing testing versus single-gene testing among patients with metastatic non-small cell lung cancer based on current Canadian practices. Curr Oncol.

[19] Zou D., Ye W., Hess L.M. (2022). Diagnostic value and cost-effectiveness of next-generation sequencing–based testing for treatment of patients with advanced/metastatic non-squamous non–small-cell lung cancer in the United States. J Mol Diagn.

[20] Pennell N.A., Mutebi A., Zhou Z.Y. (2019). Economic impact of next-generation sequencing versus single-gene testing to detect genomic alterations in metastatic non–small-cell lung cancer using a decision analytic model. JCO Precis Oncol.

[21] Arriola E., Bernabé R., Campelo R.G. (2023). Cost-effectiveness of next-generation sequencing versus single-gene testing for the molecular diagnosis of patients with metastatic non–small-cell lung cancer from the perspective of Spanish reference centers. JCO Precis Oncol.

[22] Wolff H.B., Steeghs E.M.P., Mfumbilwa Z.A. (2022). Cost-effectiveness of parallel versus sequential testing of genetic aberrations for stage IV non–small-cell lung cancer in the Netherlands. JCO Precis Oncol.

[23] Ortendahl J.D., Cuyun Carter G., Thakkar S.G., Bognar K., Hall D.W., Abdou Y. (2024). Value of next generation sequencing (NGS) testing in advanced cancer patients. J Med Econ.

[24] Ettinger D.S., Wood D.E., Aisner D.L. (2021). NCCN guidelines insights: non–small cell lung cancer, version 2.2021: featured updates to the NCCN guidelines. J Natl Compr Cancer Netw.

[25] Lindeman N.I., Cagle P.T., Aisner D.L. (2018). Updated molecular testing guideline for the selection of lung cancer patients for treatment with targeted tyrosine kinase inhibitors: guideline from the college of American pathologists, the international association for the study of lung cancer, and the association for molecular pathology. J Mol Diagn.

[26] Schilsky R.L., Longo D.L. (2022). Closing the gap in cancer genomic testing. N Engl J Med.

[27] Diamond A., Sekhon J.S. (2013). Genetic matching for estimating causal effects: a general multivariate matching method for achieving balance in observational studies. Rev Econ Stat.

[28] Sekhon J.S. (2011). Multivariate and propensity score matching software with automated balance optimization: the matching package for R. J Stat Software.

[29] Greenland S., Pearl J., Robins J.M. (1999). Causal diagrams for epidemiologic research. Epidemiology.

[30] Textor J., van der Zander B., Gilthorpe M.S., Liśkiewicz M., Ellison G.T. (2016). Robust causal inference using directed acyclic graphs: the R package ‘dagitty. Int J Epidemiol.

[31] Presley C.J., Tang D., Soulos P.R. (2018). Association of broad-based genomic sequencing with survival among patients with advanced non–small cell lung cancer in the community oncology setting. JAMA.

[32] Kang D.W., Park S.K., Yu Y.L., Lee Y., Lee D.H., Kang S. (2024). Effectiveness of next-generation sequencing for patients with advanced non-small-cell lung cancer: a population-based registry study. ESMO Open.

[33] Stencel K., Chmielewska I., Milanowski J., Ramlau R. (2021). Non-small-cell lung cancer: new rare targets—new targeted therapies—state of the art and future directions. Cancers.

[34] Grodzka A., Knopik-Skrocka A., Kowalska K. (2023). Molecular alterations of driver genes in non-small cell lung cancer: from diagnostics to targeted therapy. EXCLI J.

[35] Lundh A., Lexchin J., Mintzes B., Schroll J.B., Bero L. (2017). Industry sponsorship and research outcome. Cochrane Database Syst Rev.

[36] Xie F., Zhou T. (2022). Industry sponsorship bias in cost effectiveness analysis: registry based analysis. BMJ.

[37] Zhou T., Xie F. (2023). Sponsorship bias in oncology cost effectiveness analysis. J Clin Epidemiol.

[38] Park J.J.H., Siden E., Zoratti M.J. (2019). Systematic review of basket trials, umbrella trials, and platform trials: a landscape analysis of master protocols. Trials.

[39] Schneeweiss S. (2019). Real-world evidence of treatment effects: the useful and the misleading. Clin Pharmacol Ther.

[40] Capkun G., Corry S., Dowling O. (2022). Can we use existing guidance to support the development of robust real-world evidence for health technology assessment/payer decision-making?. Int J Technol Assess Health Care.

[41] Saldarriaga E.M., Hauber B., Carlson J.J., Barthold D., Veenstra D.L., Devine B. (2022). Assessing payers’ preferences for real-world evidence in the United States: a discrete choice experiment. Value Health.

[42] Simons M.J.H.G., Uyl-de Groot C.A., Retèl V.P. (2023). Cost-effectiveness and budget impact of future developments with whole-genome sequencing for patients with lung cancer. Value Health.

[43] Regier D.A., Weymann D., Buchanan J., Marshall D.A., Wordsworth S. (2018). Valuation of health and nonhealth outcomes from next-generation sequencing: approaches, challenges, and solutions. Value Health.

[44] Bombard Y., Baker G.R., Orlando E. (2018). Engaging patients to improve quality of care: a systematic review. Implement Sci.

[45] Regier D.A., Veenstra D.L., Basu A., Carlson J.J. (2020). Demand for precision medicine: a discrete-choice experiment and external validation study. Pharmacoeconomics.

[46] Kurian A.W., Ford J.M. (2015). Multigene panel testing in oncology practice: how should we respond?. JAMA Oncol.

[47] Regier D.A., Pollard S., McPhail M. (2022). A perspective on life-cycle health technology assessment and real-world evidence for precision oncology in Canada. NPJ Precis Oncol.

[48] Weymann D., Laskin J., Jones S.J.M. (2021). Matching methods in precision oncology: an introduction and illustrative example. Mol Genet Genomic Med.

[49] Sheffield K.M., Bowman L., Smith D.M. (2018). Development and validation of a claims-based approach to proxy ECOG performance status across ten tumor groups. J Comp Eff Res.

