# Supplementary material for: Real-world cost-effectiveness of multi-gene panel sequencing to inform therapeutic decisions for advanced non-small cell lung cancer: a population-based study
Source: Lancet Reg Health Am. 2024 Nov 15;40:100936. doi: 10.1016/j.lana.2024.100936 (PMC11599455; doi:10.1016/j.lana.2024.100936)
Supplement: Oncopanel_Supplement [file mmc1.pdf]

Supplemental Material

Real-world cost-effectiveness of multi-gene panel sequencing to inform therapeutic decisions for advanced non-small cell lung cancer: a population-based study

Emanuel Krebs [1], Deirdre Weymann [1], Cheryl Ho [2,3], Ian Bosdet [4,5], Janessa Laskin [2,3], Howard J Lim [2,3], Stephen Yip [4,5], Aly Karsan [4,6], Timothy P Hanna [7,8], Samantha Pollard [1], Dean A Regier [1,9]

1. Cancer Control Research, BC Cancer Research Institute, Vancouver, BC, Canada. 2. Department of Medical Oncology, BC Cancer, Vancouver, BC, Canada. 3. Department of Medicine, Faculty of Medicine, University of British Columbia, Vancouver, BC, Canada. 4. Department of Pathology & Laboratory Medicine, Faculty of Medicine, University of British Columbia, Vancouver, BC, Canada. 5. Cancer Genetics & Genomics Laboratory, BC Cancer, Vancouver, BC, Canada. 6. Michael Smith Genome Sciences Centre, BC Cancer Research Institute, Vancouver, BC, Canada. 7. Department of Oncology, Queen's University, Kingston, ON, Canada 8. Department of Public Health Science, Queen's University, Kingston, ON, Canada. 9. School of Population and Public Health, Faculty of Medicine, University of British Columbia; Vancouver, BC, Canada.

S1. Administrative health databases, covariates and matching.

Administrative health databases.

Population Data BC serves as a multi-university data resource, employing both deterministic and probabilistic linkage techniques to ensures broad coverage of health data for almost all residents in BC [1].

BC Cancer databases included the BC Cancer Registry (demographic and disease information at primary cancer diagnosis, and mortality), the BC Cancer Genetics and Genomics Laboratory database (single-gene and multi-gene panel test records, results and costs), the BC Cancer Pharmacy database (systemic therapy type, date of administration, protocol code, and billed drug costs for publicly funded systemic therapy drugs), the Radiation database (external beam radiation therapy and brachytherapy, including the total number of fractions), surgery (cancer-specific surgical procedures) and the Cancer Agency Information System (CAIS) scheduling database (appointments for consultations with oncologists and ambulatory care services delivered at BC Cancer sites). Population Data BC databases included PharmaNet (capturing all drug dispensations and costs outside of BC's acute care hospitals) [2], the Medical Services Plan (MSP; costs of fee for service physician services and diagnostic tests) [3,4], and the Discharge Abstract Database (DAD; costs and utilization of inpatient care and day surgeries) [5]. Health administrative databases were linked using unique anonymized patient identifiers.

Covariates and matching.

Sociodemographic covariates accounted for sex at birth, time since diagnosis, age at diagnosis, provincial health authority of residence, and rural or remote area of residence (as defined by the province in the planning and reporting of health services [6]).

Clinical covariates included number of lines of systemic therapy received prior to the index date, total number of primary cancer sites identified for each patient, any history of surgical treatment or palliative radiotherapy, a modified version of the Charlson Comorbidity Index (CCI) calculated for the 12 months prior to the index date and derived using ICD-10 codes from hospitalization records that excluded cancer-specific categories [7], a chronic disease score (CDS) based on dispensation records of 29 non-cancer prescription medication categories calculated for the six months prior to the index date [8], any history of metastasis-related hospitalization in the 12 months prior to the index date, and costs of health resource use in the month prior to the index date.

To determine the number of lines of systemic therapy received by each patient prior to the index date, we applied an automated algorithm validated for use in BC Cancer's administrative systemic therapy data [9].

Sociodemographic covariates used for propensity score estimation and for matching included sex, time since diagnosis, age at diagnosis, and area of residence. Clinical covariates used for propensity score estimation and for matching included number of prior lines of systemic therapy, number of cancer sites, prior costs of health resource use, and indicator variables for prior surgical treatment, prior palliative radiotherapy, prior hospitalization, a modified Charlson Comorbidity Index score greater than zero, and a chronic disease score greater than sex-specific median values.

Figure. Directed acyclic graphs (DAGs) informed by discussion with clinicians to identify the minimal sufficient covariate adjustment set necessary for estimating the total effect of multi-gene panel sequencing on survival [10,11].

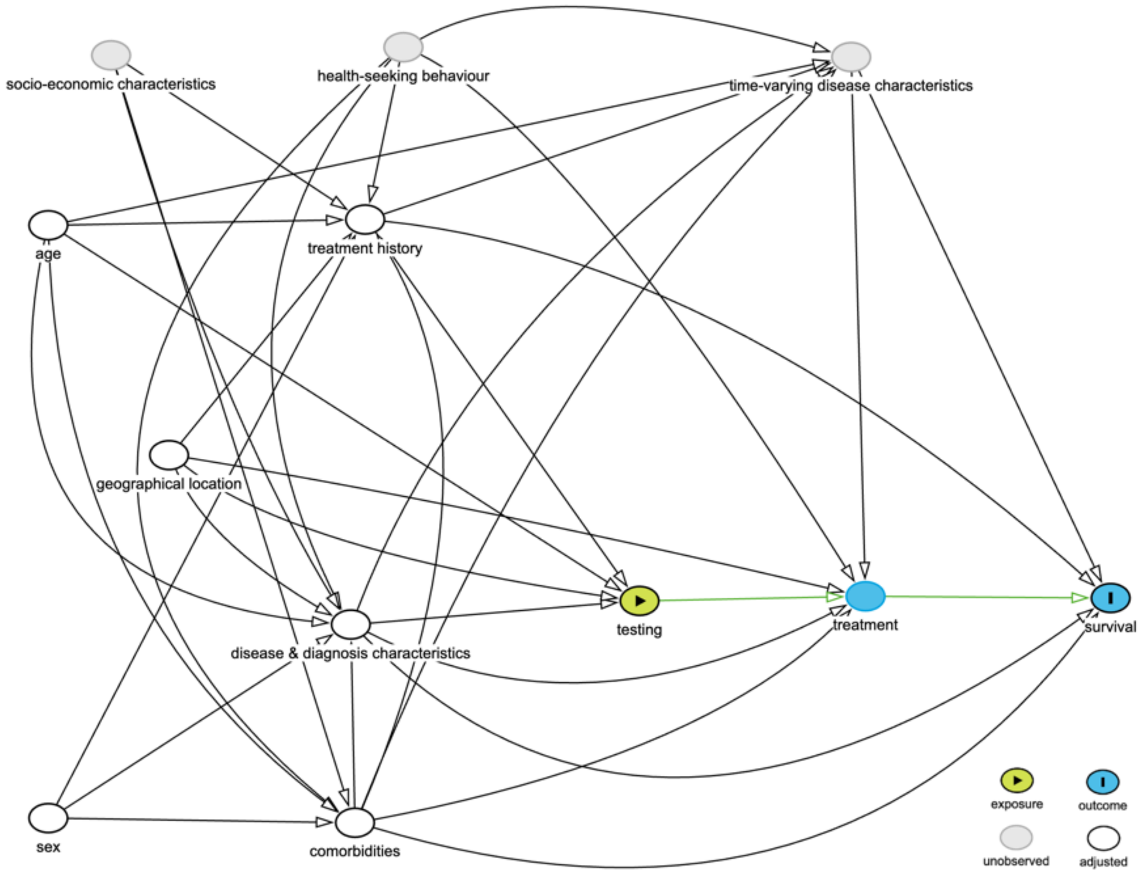

S2. Cancer care costs.

In BC, systemic therapy treatments administered in regional cancer centers, community hospitals, or taken at home are dispensed by BC Cancer pharmacies, and records capture billed drug costs for publicly funded systemic therapy drugs. Given that BC Cancer care services are not delivered on a fee-for-service basis, we obtained corresponding unit costs from published fees for similar services, literature sources, and/or expert opinion, as previously detailed [12].

S3. Covariate balance before and after matching. *

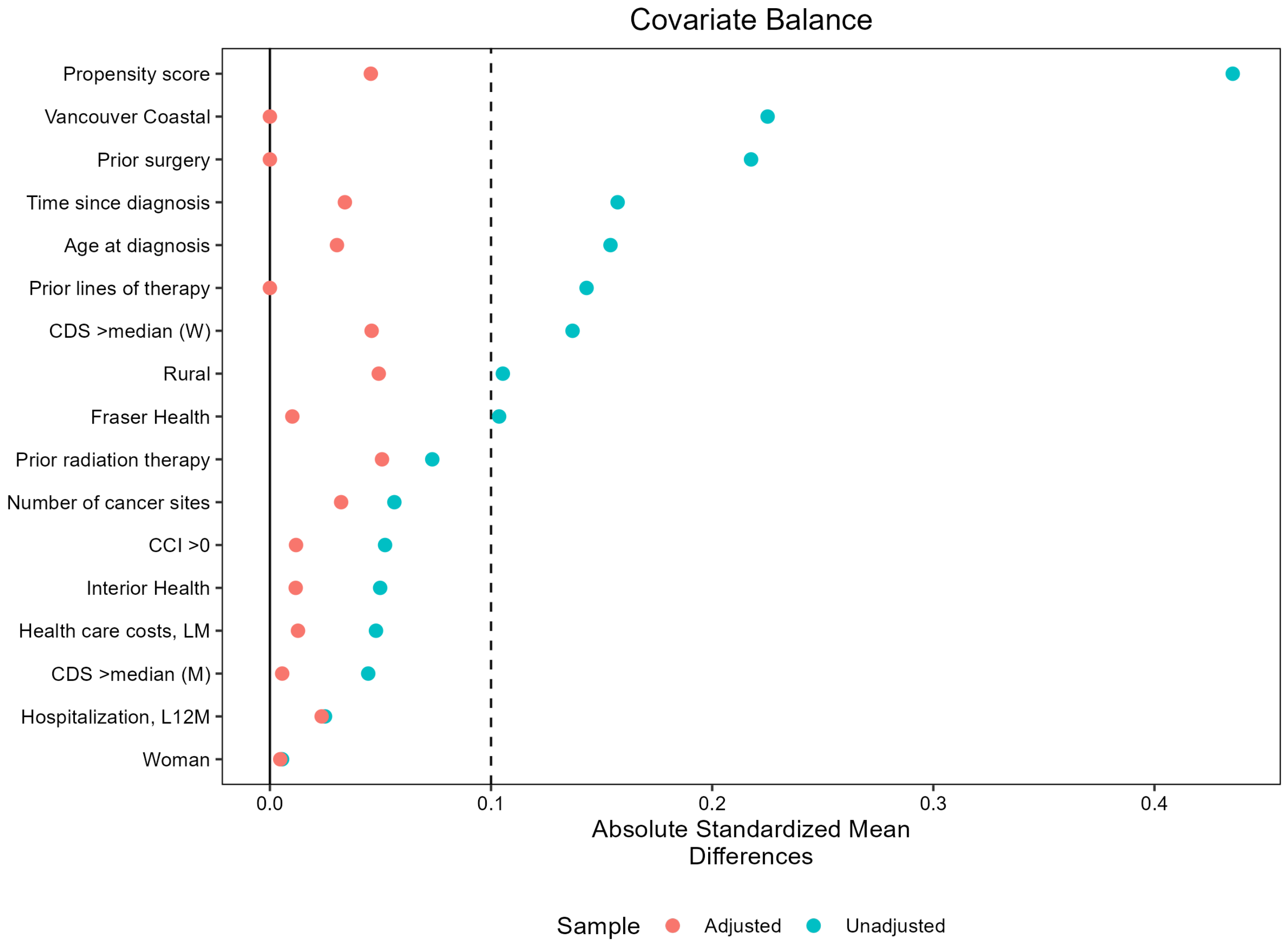

* We matched patients receiving panel-based testing 1:1 with control patients on covariates indicated above, exact matching on number of prior lines of therapy, history of surgical treatment, and residency in the Vancouver Coastal Health Authority, allowing for ties and matching without replacement.

S4. Quantile-Quantile (QQ) plot for time since diagnosis.

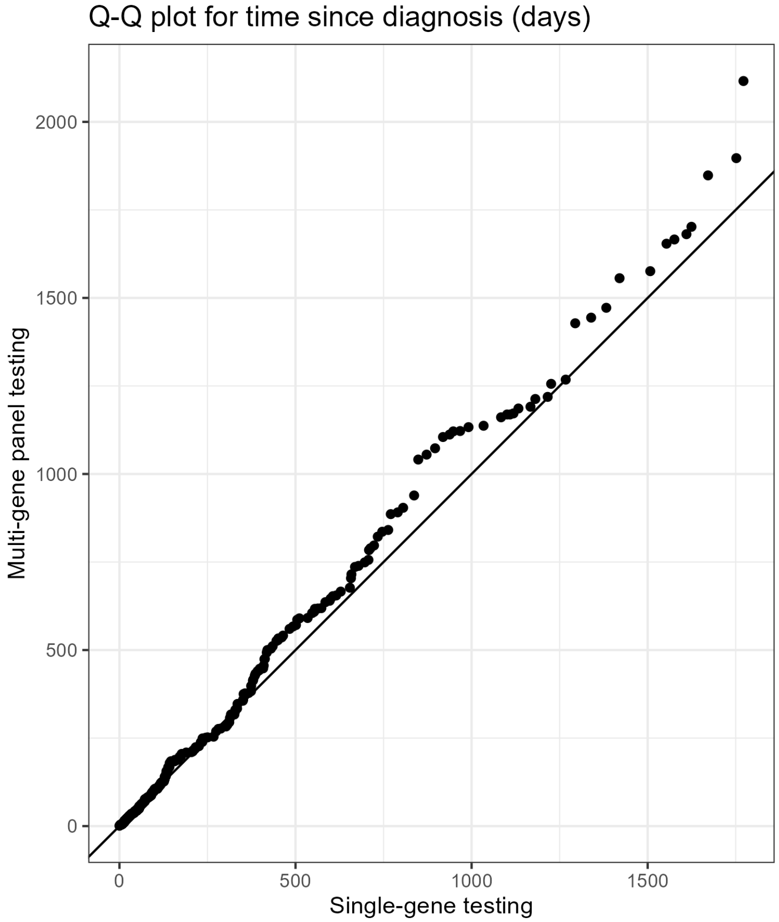

S5. Test results and time from specimen collection to lab receipt, before and after matching.

A. Test results before matching.

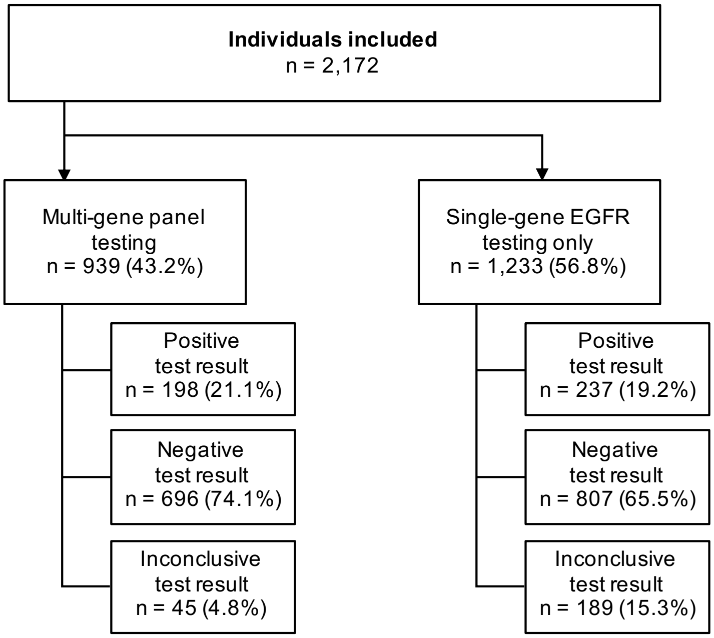

B. Test results after matching.

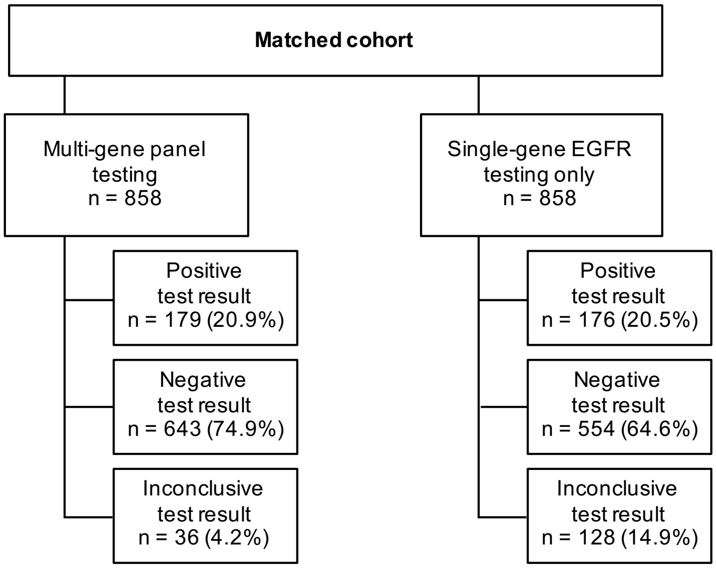

Descriptive statistics on time from specimen collection to lab receipt for testing, before and after matching:

- mean times were statistically different in unmatched patients (87 days for single-gene testing vs 136 days for multi-gene panels; $p<0.001$)
- mean times were <u>not statistically different</u> in matched patients (94 days for single-gene testing vs 114 days for multi-gene panels; $p=0.126$)
- median number of days are the same across testing groups, both in matched and unmatched patients (28 days)

S6. Overall survival for multi-gene panel testing compared to single-gene EGFR testing in the matched cohort. *

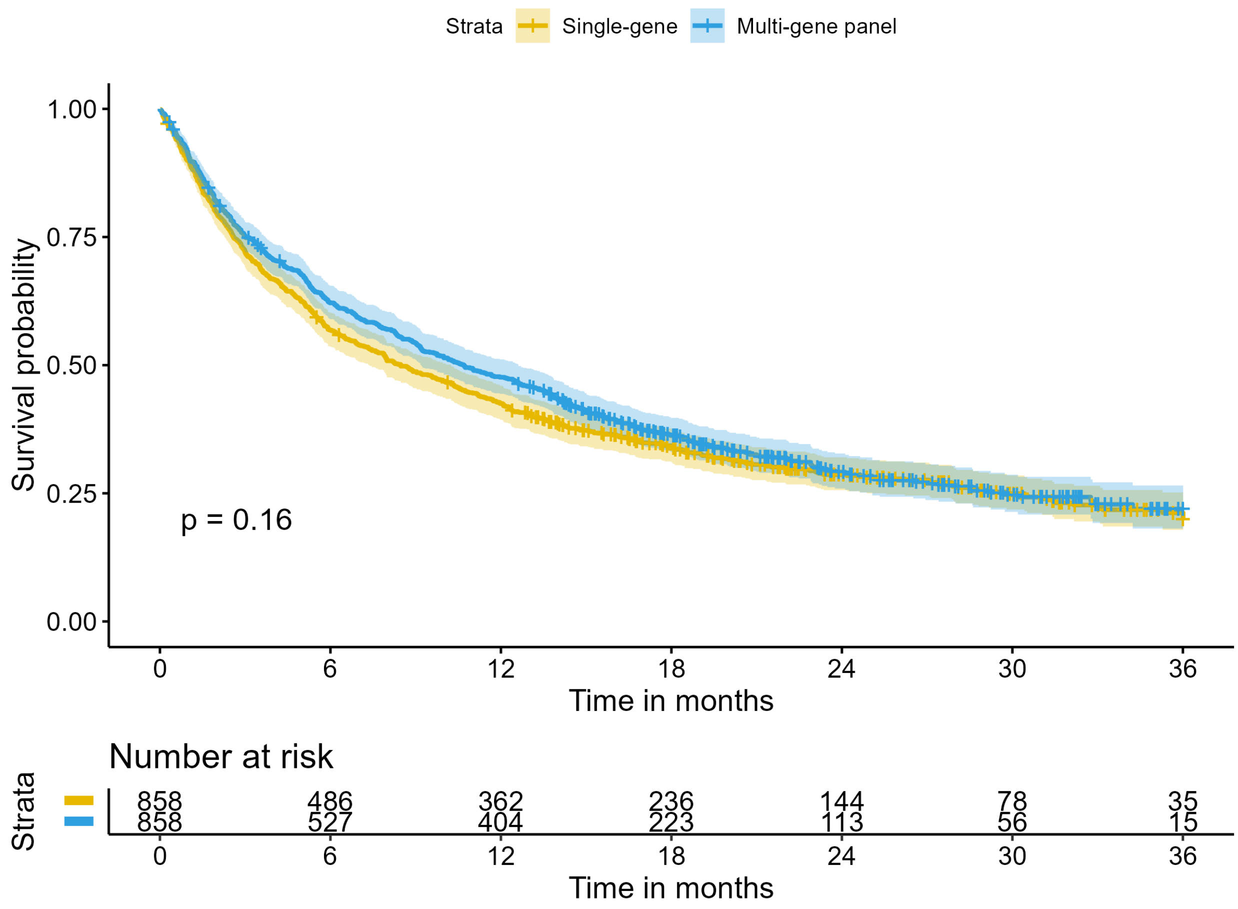

* Reported p-value is from log-rank test.

S7. Mean and median survival Kaplan-Meier estimates* for multi-gene panel testing or single-gene EGFR testing, in days.

	Mean (95% CI)*		Median (95% CI)	
	Single-gene	Multi-gene panel	Single-gene	Multi-gene panel
	n (%)	n (%)	n (%)	n (%)
**Base case**	442 (414, 471)	469 (440, 497)	258 (223, 306)	325 (279, 384)
Sensitivity analysis				
*Matching with replacement^*	442 (414, 471)	484 (456, 512)	258 (223, 306)	336 (293, 400)
Matching without VCH	450 (422, 479)	478 (449, 506)	269 (231, 321)	332 (281, 387)
Matching with Interior Health	451 (423, 480)	476 (448, 505)	270 (231, 321)	327 (281, 387)
Matching with Fraser Health	442 (414, 471)	475 (447, 504)	258 (223, 312)	326 (280, 384)
Positive test results	635 (571, 700)	624 (561, 686)	614 (509, 929)	594 (504, 750)
Negative test results	391 (357, 425)	429 (397, 462)	193 (165, 242)	260 (216, 297)

* Mean survival times were calculated as the area under the Kaplan–Meier survivor functionare with estimates that are restricted to the longest follow-up time.

^ Matching with replacement was restricted to using each control a maximum of three times.

StataCorp. 2019. Stata 16 Survival Analysis Reference Manual. College Station, TX: Stata Press

S8. Cumulative average costs by category for each testing strategy, over the study period and by year.

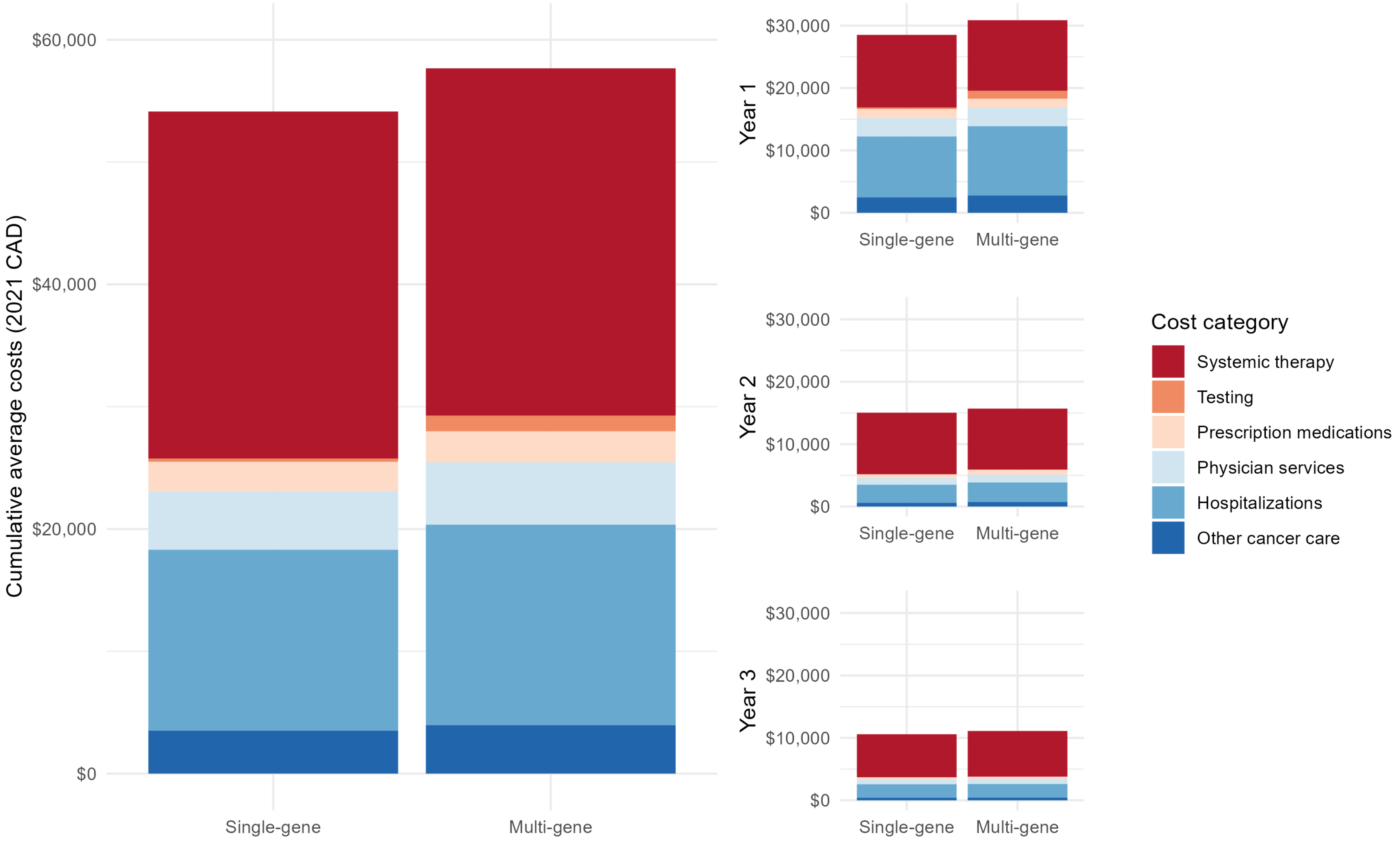

S9. Cost-effectiveness of multi-gene panel testing compared to single-gene EGFR testing estimated using seemingly unrelated regressions (SUR).

The seemingly unrelated regressions (SUR) estimation considered weighted costs and survival time based on the inverse probability of being observed at the start of each monthly interval to account for censoring arising from incomplete follow-up data. We estimated mean three-year survival time and costs with the system of linear monthly equations for survival time and costs allowing for errors to be correlated across equations for each patient but uncorrelated across patients.

	Treated (*n*)*	Incremental life-years gained (LYG)	Incremental total cost, 2021 CAD
SUR	858	0.02 [-0.00, 0.03]	$2,203 [-$444, $4,849]

* Number of patients included in the analysis after 1:1 matching to controls.

S10. Impact inventory.

Sector	Type of Impact	Perspective		Notes
		Public healthcare payer	Healthcare	
**Formal Health Care**				
Health	**Health Outcomes (Effects)**			
	Longevity	√	√	
	HRQoL			
	Other Health Effects			
	**Medical Costs**			
	Third-Party Payers	√	√	
	Patients out-of-pocket		√	Prescription drugs
	Future related medical costs	√	√	
	Future unrelated medical costs			
**Informal Health Care**				
Health	Patient-time costs	N/A		
	Unpaid caregiver-time costs	N/A		
	Transportation costs	N/A		
**Non-Health Care Sectors**				
Productivity	Labour market earnings lost	N/A		
	Cost of unpaid lost productivity	N/A		
	Cost of uncompensated household production	N/A		
Consumption	Future consumption unrelated to health	N/A		
Social Services	Cost of social services related to intervention	N/A		
Legal or criminal justice	Number of crimes related to intervention	N/A		
	Cost of crimes related to intervention	N/A		
Education	Impact on educational achievement	N/A		
Housing	Cost of intervention on home improvements	N/A		
Environment	Production of toxic waste by intervention	N/A		
Other	Other impacts	N/A		

HRQoL: Health-related quality of life; Impact Inventory included on recommendation from the Second Panel on Cost-Effectiveness in Health and Medicine. Neumann, Peter J., Gillian D. Sanders, Louise B. Russell, Joanna E. Siegel, and Theodore G. Ganiats. *Cost-Effectiveness in Health and Medicine (2nd Edn)*. Oxford University Press, 2016. https://academic.oup.com/book/12265.

S11. Reporting checklist.

Element	Journal Article	Supplement
**Introduction**		
Background of the problem	√	
**Study Design and Scope**		
Objectives	√	
Audience	√	
Type of analysis	√	
Target population	√	
Description of intervention and comparators	√	
Other intervention descriptors	√	
Boundaries of the analysis; defining the scope or comprehensiveness of the study	√	
Time horizon	√	
Analytic perspectives	√	
Whether this analysis meets the requirements of the reference case	√	
Analysis plan	√	
**Methods and data**		
If model based analysis:		
Description of event pathway or model		
Model diagram		
Description of the model used		
Modeling assumptions		
Software used		
Identification of key outcomes	√	
Complete information on sources of effectiveness data, cost data, and preference weights	√	
Methods for obtaining estimates of effectiveness (including approaches used for evidence synthesis)	√	
Methods for obtaining estimates of costs and preference weights	√	
Critique of data quality	√	
Statement of costing year	√	
Statement of methods used to adjust for inflation	√	
Statement of currency used	√	
Source of expert judgement (if applicable)	√	
Statement of discount rates	√	
**Impact Inventory**		
Full accounting of consequences within and outside the health care sector		√
**Results**		
Results of model validation		N/A
Reference case results: total costs and effectiveness, incremental costs and effectiveness, ICERs, uncertainty measures	√	
Disaggregated results for important categories of costs, outcomes or both	√	
Results of sensitivity analysis	√	√
Other estimates of uncertainty	√	√
Graphical representation of cost-effectiveness results		√
Graphical representation of uncertainty analyses	√	√
Aggregate cost and effectiveness information	√	
Secondary analyses	√	√
**Disclosures**		
Statement of any potential conflicts of interest due to funding source, collaborations, or outside interests	√	
**Discussion**		
Summary of reference case results	√	

Summary of sensitivity of results to assumptions and uncertainties in the analysis	√	
Discussion of the study results in the context of results of related cost-effectiveness analyses	√	
Discussion of ethical implications	√	
Limitations of the study	√	
Relevance of study results to specific policy questions or decisions	√	

Reporting Checklist included on recommendation from the Second Panel on Cost-Effectiveness in Health and Medicine. Neumann, Peter J., Gillian D. Sanders, Louise B. Russell, Joanna E. Siegel, and Theodore G. Ganiats. *Cost-Effectiveness in Health and Medicine (2nd Edn)*. Oxford University Press, 2016. https://academic.oup.com/book/12265.

S12. CHEERS checklist.

Item	Item	Recommendation	Section
**Title**	1	Identify the study as an economic evaluation and specify the interventions being compared.	Title
**Abstract**	2	Provide a structured summary that highlights context, key methods, results, and alternative analyses.	Abstract
**Introduction**			
Background and objectives	3	Give the context for the study, the study question, and its practical relevance for decision making in policy or practice.	Introduction
**Methods**			
Health economic analysis plan	4	Indicate whether a health economic analysis plan was developed and where available.	Paragraphs 7-9 in Methods
Study population	5	Describe characteristics of the study population (such as age range, demographics, socioeconomic, or clinical characteristics).	Paragraph 1 in Methods
Setting and location	6	Provide relevant contextual information that may influence findings.	Paragraph 1 in Methods
Comparators	7	Describe the interventions or strategies being compared and why chosen.	Paragraph 3 in Methods
Perspective	8	State the perspective(s) adopted by the study and why chosen.	Paragraphs 7-9 in Methods
Time horizon	9	State the time horizon for the study and why appropriate.	Paragraphs 1, 7 & 9 in Methods
Discount rate	10	Report the discount rate(s) and reason chosen.	Paragraph 7 in Methods
Selection of outcomes	11	Describe what outcomes were used as the measure(s) of benefit(s) and harm(s).	Paragraph 7 in Methods
Measurement of outcomes	12	Describe how outcomes used to capture benefit(s) and harm(s) were measured.	Paragraphs 2, 3 & 7 in Methods
Valuation of outcomes	13	Describe the population and methods used to measure and value outcomes.	Paragraphs 7-9 in Methods
Measurement and valuation of resources and costs	14	Describe how costs were valued.	Paragraphs 8 in Methods
Currency, price date, and conversion	15	Report the dates of the estimated resource quantities and unit costs, plus the currency and year of conversion.	Paragraphs 7-9 in Methods
Rationale and description of model	16	If modelling is used, describe in detail and why used. Report if the model is publicly available and where it can be accessed.	N/A
Analytics and assumptions	17	Describe any methods for analysing or statistically transforming data, any extrapolation methods, and approaches for validating any model used.	Paragraphs 4-6 & 9 in Methods
Characterising heterogeneity	18	Describe any methods used for estimating how the results of the study vary for subgroups.	Paragraph 10 in Methods
Characterising distributional effects	19	Describe how impacts are distributed across different individuals or adjustments made to reflect priority populations.	N/A
Characterising uncertainty	20	Describe methods to characterise any sources of uncertainty in the analysis.	Paragraph 9 in Methods
Approach to engagement with patients and others affected by the study	21	Describe any approaches to engage patients or service recipients, the general public, communities, or stakeholders (such as clinicians or payers) in the design of the study.	N/A
**Results**			
Study parameters	22	Report all analytic inputs (such as values, ranges, references) including uncertainty or distributional assumptions.	Paragraphs 1-4 in Results
Summary of main results	23	Report the mean values for the main categories of costs and outcomes of interest and summarise them in the most appropriate overall measure.	Paragraph 5 in Results

Effect of uncertainty	24	Describe how uncertainty about analytic judgments, inputs, or projections affect findings. Report the effect of choice of discount rate and time horizon, if applicable.	Paragraphs 5-7 in Results
Effect of engagement with patients and others affected by the study	25	Report on any difference patient/service recipient, general public, community, or stakeholder involvement made to the approach or findings of the study	N/A
Discussion			
Findings, limitations, generalizability, and current knowledge	22	Summarize key findings and describe how they support the conclusions reached, and limitations to generalizability	Discussion
Other relevant information			
Source of funding	23	Describe study funding and other non-monetary sources of support	Role of the Funding Source
Conflicts of interest	24	Describe any potential conflicts of interest	Author Funding/Conflict Statements

Source: Husereau D, Drummond M, Augustovski F, et al. Consolidated Health Economic Evaluation Reporting Standards 2022 (CHEERS 2022) Explanation and Elaboration: A Report of the ISPOR CHEERS II Good Practices Task Force. Value Health 2022;25.

References

1. Ark TK, Kesselring S, Hills B, McGrail K. Population Data BC: Supporting population data science in British Columbia. International Journal of Population Data Science [Internet]. 2019 [cited 2024 May 14];4(2). Available from: https://ijpds.org/article/view/1133

2. British Columbia Ministry of Health [creator] (2021). PharmaNet. V2. Population Data BC [publisher]. Data Extract. Data Stewardship Committee (2021). http://www.popdata.bc.ca/data.

3. British Columbia Ministry of Health [creator] (2021). Medical Services Plan (MSP) Payment Information File. V2. Population Data BC [publisher]. Data Extract. MOH (2021). http://www.popdata.bc.ca/data.

4. British Columbia Ministry of Health [creator] (2021). Consolidation File (MSP Registration & Premium Billing). V2. Population Data BC [publisher]. Data Extract. MOH (2021). http://www.popdata.bc.ca/data.

5. Canadian Institute for Health Information [creator] (2021). Discharge Abstract Database (Hospital Separations). V2. Population Data BC [publisher]. Data Extract. MOH (2021). http://www.popdata.bc.ca/data.

6. Health Sector Information, Analysis and Reporting (HSIAR) Division. B.C. Health System Strategy, Geographic Service Areas [Internet]. British Columbia Ministry of Health; 2016. Available from: www2.gov.bc.ca/assets/gov/health/about-bc-s-health-care-system/health-priorites/geographic-service-areas.docx&usg=AOvVaw0CyCd-eYodYcuhskT6UMjL

7. Quan H, Sundararajan V, Halfon P, Fong A, Burnand B, Luthi JC, et al. Coding Algorithms for Defining Comorbidities in ICD-9-CM and ICD-10 Administrative Data. Medical Care. 2005 Nov;43(11):1130–9.

8. Clark DO, Korff MV, Saunders K, Baluch WM, Simon GE. A Chronic Disease Score with Empirically Derived Weights. Medical Care. 1995 Aug;33(8):783–95.

9. Weymann D, Costa S, Regier DA. Validation of a Cyclic Algorithm to Proxy Number of Lines of Systemic Cancer Therapy Using Administrative Data. JCO Clinical Cancer Informatics. 2019 Dec;(3):1–10.

10. Greenland S, Pearl J, Robins JM. Causal Diagrams for Epidemiologic Research. Epidemiology. 1999 Jan;10(1):37–48.

11. Textor J, van der Zander B, Gilthorpe MS, Liśkiewicz M, Ellison GT. Robust causal inference using directed acyclic graphs: the R package 'dagitty.' International Journal of Epidemiology. 2016 Dec 1;45(6):1887–94.

12. Weymann D, Laskin J, Jones SJM, Roscoe R, Lim HJ, Renouf DJ, et al. Early-stage economic analysis of research-based comprehensive genomic sequencing for advanced cancer care. J Community Genet [Internet]. 2021 Nov 29 [cited 2022 Aug 15]; Available from: https://doi.org/10.1007/s12687-021-00557-w
